# Epigenetic mortality predictors and incidence of breast cancer

**DOI:** 10.18632/aging.102523

**Published:** 2019-12-17

**Authors:** Jacob K. Kresovich, Zongli Xu, Katie M. O’Brien, Clarice R. Weinberg, Dale P. Sandler, Jack A. Taylor

**Affiliations:** 1 Epidemiology Branch, National Institute of Environmental Health Sciences, NIH, Durham, NC 27709, USA; 2 Biostatistics and Computation Biology Branch, National Institute of Environmental Health Sciences, NIH, Durham, NC 27709, USA; 3 Epigenetic and Stem Cell Biology Laboratory, National Institute of Environmental Health Sciences, NIH, Durham, NC 27709, USA

**Keywords:** breast cancer, DNA methylation, epigenetic clock, mortality score, grimAge

## Abstract

Measures derived using blood DNA methylation are increasingly under investigation as indicators of disease and mortality risk. Three existing epigenetic age measures or “epigenetic clocks” appear associated with breast cancer. Two newly-developed epigenetic mortality predictors may be related to all-cancer incidence, but associations with specific cancers have not been examined in large studies. Using HumanMethylation450 BeadChips to measure blood DNA methylation in 2,773 cancer-free women enrolled in the Sister Study, we calculated two epigenetic mortality predictors: ‘GrimAgeAccel’ and the ‘mortality score’ (MS). Using Cox proportional hazard models, neither GrimAgeAccel nor the MS were associated with overall breast cancer incidence (GrimAgeAccel hazard ratio [HR]: 1.06, 95% confidence interval [CI]: 0.98-1.14, *P*=0.17; MS HR: 0.99, 95% CI: 0.92-1.07, *P*=0.85); however, a weak, positive association was observed for GrimAgeAccel and invasive breast cancer (HR: 1.08, 95% CI: 0.99-1.17, *P*=0.08). Stratification of invasive cancers by menopause status at diagnoses revealed the association was predominantly observed for postmenopausal breast cancer (HR: 1.10, 95% CI: 1.01, 1.20, *P*=0.04). Although the MS was unrelated to breast cancer risk, we find evidence that GrimAgeAccel may be weakly associated with invasive breast cancer, particularly for women diagnosed after menopause.

## INTRODUCTION

Age related changes to blood DNA methylation (DNAm) have been extensively reported [1–4] and serve as the foundation for epigenetic clocks [5–7]. These clocks were designed to predict the rate of aging and may reflect underlying aging processes [8], including transcriptional regulation and cellular differentiation [9, 10]. Compared to other molecular markers of aging, those developed using DNAm appear particularly robust [11]. To date, at least three epigenetic clocks have been constructed. These clocks comprise hundreds of CpGs and are reported to be associated with the incidence of chronic diseases and mortality [12–20], as well as specific cancers [21–24], including breast cancer [25].

While epigenetic clocks were designed to predict the biological consequences of aging, other combinations of CpGs have been identified to predict mortality [26, 27]. GrimAgeAccel, a new DNAm-based mortality predictor, was recently reported to be associated with time-to-death [26]. Using genome-wide DNAm data from the Framingham Heart Study (FHS; N= 2,356 individuals), GrimAgeAccel was constructed as a composite biomarker using a multistep approach: in the first step, various DNAm-predictors were separately developed for smoking pack-years and for a number of mortality-associated plasma proteins; then, these DNAm-predictors were used, along with self-reported sex and age, in an elastic net Cox regression trained on all-cause mortality to derive ‘DNAm GrimAge;’ finally, using a linear model, GrimAgeAccel was defined as the raw residuals from regressing DNAm GrimAge on chronological age. GrimAgeAccel was validated as an epigenetic mortality predictor across five, racially-diverse cohorts (N= 6,935) [26].

GrimAgeAccel was previously reported to be associated with all-cancer incidence [26]. Using DNAm data from the FHS (446 participants, 40 cancer cases) and the Women’s Health Initiative (WHI; 4,079 participants, 730 cancer cases), GrimAgeAccel was associated with cancer incidence in the WHI but not the FHS [26]. Information on the types of cancer represented were not presented, however, the most common cancer for the WHI demographic is postmenopausal breast cancer [21, 28].

The ‘mortality score’ (MS), a separate DNAm-based predictor, was also designed to predict all-cause mortality [27]. Using 58 validated, mortality-associated CpGs identified in the ESTHER cohort (N= 1,954), the MS was constructed by selecting an informative set of these CpGs using LASSO Cox regression trained on all-cause mortality. The MS was then validated across the ESTHER (N= 1,000) and KORA (N= 1,727) cohorts. Although the MS was originally developed as an ordinal score based on the proportion of aberrantly methylated CpGs, a continuous version was calculated as a linear combination of CpGs where the regression coefficients served as weights [27]. An independent study from the Normative Aging Study (N= 534) suggested the continuous version of the MS was a stronger predictor of all-cause mortality [29].

Although GrimAgeAccel and the MS were designed to predict all-cause mortality, they may be useful in predicting cancer incidence. In additional to being strongly associated with all-cancer incidence, GrimAgeAccel may also predict menopause timing [26]; the MS is associated with cancer mortality [27], and may also be a marker of cancer incidence. To date, however, neither GrimAgeAccel nor the MS has been examined in relation to incidence of specific cancers. Using a large, nationwide, prospective cohort established to study breast cancer, we find evidence that GrimAgeAccel, but not the MS, may be weakly related to invasive breast cancer incidence, particularly for women diagnosed after menopause.

## RESULTS

**Figure 1:**
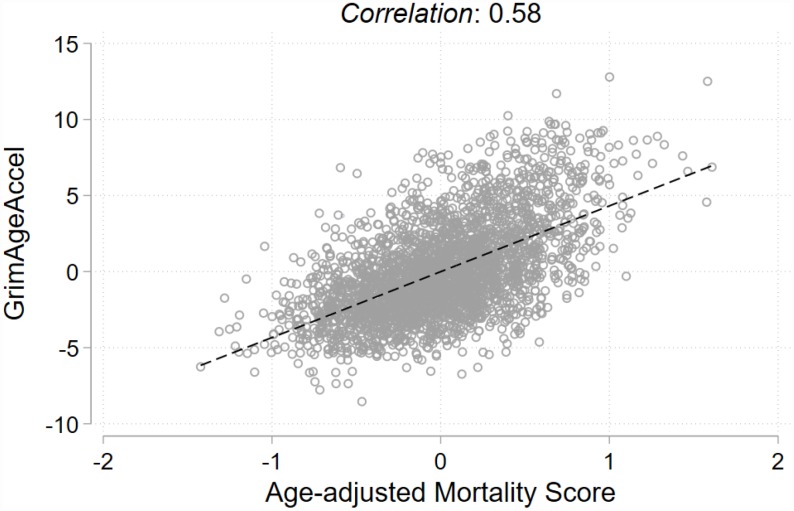
**Pearson correlation and fit line for the two epigenetic mortality predictors.**

At enrollment, our case-cohort population of non-Hispanic white women (N= 2,773) had an average age of 56.6 years (standard deviation [SD]= 8.8 years); 70% of whom reported being postmenopausal. The women who developed breast cancer tended to be older at enrollment, drank more alcohol, were less physically active, had later ages at menopause, and were more likely to report postmenopausal hormone use (Table 1). Distributions of the continuous characteristics by cancer status at follow-up are displayed in Supplementary Figure 1. For all participants, mean follow-up was 6.0 years (SD=3.2 years) and among the cases, mean time-to-diagnosis was 3.9 years (SD=2.2 years). Most breast cancers were diagnosed as invasive (79%) and the invasive tumors were predominately estrogen receptor (ER)-positive (86%). Because the MS was positively correlated with chronological age (ρ= 0.28, *P*< 0.001), an age-adjusted MS was derived by regressing the MS on chronological age and predicting the residuals. The calculated residuals represent a MS that is independent of chronological age; as expected, this score and GrimAgeAccel were not correlated with chronological age (age-adjusted MS: ρ= -0.00, *P*= 0.99; GrimAgeAccel: ρ= -0.01, *P*= 0.61) (Supplementary Figure 2). However, GrimAgeAccel and the age-adjusted MS were positively correlated with each other (ρ= 0.58, *P*< 0.001) (Figure 1).

**Table 1:** Participant characteristics at study enrollment.

Characteristic^*^	**Cancer status at follow-up**	
	**Non-event**	**Event**
Total, N (%)	1,204 (100)	1,569 (100)
Age, mean (SD), yrs.	55.1 (9)	57.7 (9)
Alcohol, mean (SD), drinks/wk.	2.9 (4)	3.3 (5)
Physical activity, mean (SD), METs/wk.	52.4 (32)	49.6 (30)
Parity, mean (SD), total births	2.0 (1)	1.9 (1)
Age first birth, mean (SD), yrs.^1^	24.7 (5)	25.0 (5)
Menarche age, mean (SD), yrs.	12.6 (2)	12.6 (1)
Menopause age, mean (SD), yrs.^2^	49.6 (6)	50.7 (5)
GrimAgeAccel, mean (SD), yrs.	-0.1 (3)	0.0 (3)
Age-adjusted Mortality Score, mean (SD) units	0.0 (0.4)	-0.0 (0.4)
BMI, kg/m^2^, N (%)		
Underweight/normal (≤ 24.9)	482 (40)	591 (38)
Overweight (25-30)	384 (32)	515 (33)
Obese (30+)	336 (28)	463 (29)
Missing	2	0
Smoking status, N (%)		
Never	637 (53)	809 (52)
Former	475 (39)	649 (41)
Current	92 (8)	111 (7)
Oral Contraception use, N (%)		
Never	181 (15)	272 (17)
Ever	1,022 (85)	1,296 (83)
Missing	1	1
Menopause status, N (%)		
Premenopausal	408 (34)	418 (27)
Postmenopausal	795 (66)	1,151 (73)
Missing	1	0
Postmenopausal hormone use^2^, N (%)		
Never	291 (37)	372 (32)
Ever	502 (63)	775 (68)
Missing	2	4
Stage at diagnosis, N (%)		
DCIS (0)	------	338 (21)
Invasive (1-4)	------	1,231 (79)
Estrogen receptor status (invasive tumors), N (%)		
Positive	------	1,043 (86)
Negative	------	168 (14)
Missing		20

^1^Among parous women (n= 2,256)

^2^Among postmenopausal women (n= 1,946; 25 reported postmenopausal status but not age at menopause)

^*^Missing characteristics: alcohol intake, 6; physical activity, 22; parity, 2; age at first birth, 5; menarche age, 3.

Abbreviations: standard deviation, SD; metabolic equivalent task, MET; body mass index, BMI; ductal carcinoma in situ, DCIS.

**Figure 2:**
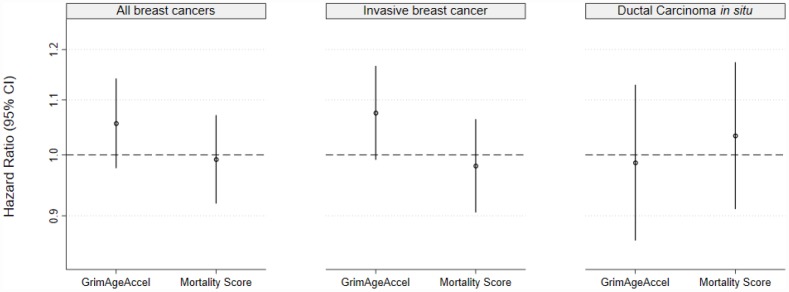
Epigenetic mortality predictors associations with breast cancer incidence for (**A**) invasive breast cancer and ductal carcinoma *in situ* (DCIS) combined and for (**B**) invasive breast cancer and (**C**) DCIS, separately.

For all breast cancers (invasive breast cancer and ductal carcinoma *in situ* [DCIS] combined), in unadjusted models, neither of the epigenetic mortality predictors were associated with breast cancer incidence (per 1-SD increase: GrimAgeAccel hazard ratio [HR]: 1.06, 95% confidence interval [CI]: 0.98, 1.14, *P*= 0.17; age-adjusted MS HR: 0.99, 95% CI: 0.92, 1.07, *P*= 0.85) (Figure 2A). In analyses stratified by invasive cancer or DCIS, a one-SD increase in GrimAgeAccel was weakly, positively associated with invasive breast cancer incidence (HR: 1.08, 95% CI: 0.99, 1.17, *P*=0.08) (Figure 2B). However, after accounting for breast cancer risk factors, the association was attenuated (adjusted HR: 1.04, 95% CI: 0.95, 1.14, *P*=0.41) (Supplementary Table 1). The age-adjusted MS was not associated with invasive breast cancer incidence (HR: 0.98, 95% CI: 0.91, 1.06, *P*=0.65) and neither the GrimAgeAccel nor age-adjusted MS metrics were associated with DCIS (GrimAgeAccel HR: 0.99, 95% CI: 0.86, 1.13, *P*=0.85; age-adjusted MS HR: 1.03, 95% CI: 0.91, 1.17, *P*=0.61) (Figure 2C).

**Figure 3:**
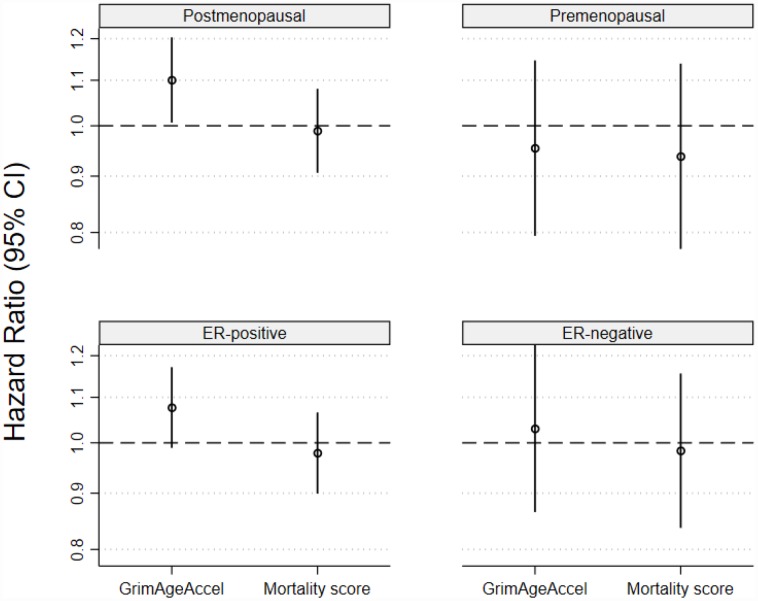
Associations for the two epigenetic mortality predictors and invasive breast cancer incidence for (**A**) postmenopausal breast cancer, (**B**) premenopausal breast cancer, (**C**) estrogen receptor positive tumors and (**D**) estrogen receptor negative tumors.

When associations were estimated for invasive breast cancers stratified by menopausal status at diagnosis or tumor ER status, in unadjusted models, a one-SD increase in GrimAgeAccel, but not the age-adjusted MS, was associated with higher postmenopausal invasive breast cancer incidence (GrimAgeAccel HR: 1.10, 95% CI: 1.01, 1.20, *P*=0.04; age-adjusted MS HR: 0.99, 95% CI: 0.91, 1.08, *P*=0.82) (Figure 3A). Accounting for breast cancer risk factors attenuated the GrimAgeAccel association (Supplementary Table 2). Neither of the epigenetic mortality predictors were associated with premenopausal invasive breast cancer (Figure 3B). A weak, positive association was observed in unadjusted models for GrimAgeAccel and invasive ER-positive tumors (HR: 1.08, 95% CI: 0.99, 1.17, *P*=0.09) (Figure 3C); model adjustment for breast cancer risk factors again shifted the association towards the null (HR: 1.04, 95% CI: 0.95, 1.14, *P*=0.40). No meaningful associations were observed for either epigenetic mortality predictor and invasive ER-negative tumors (Figure 3D).

As a post-hoc analysis to further interrogate the GrimAgeAccel metric, the separate DNAm-predictors that comprise DNAm GrimAge were tested with breast cancer overall and stratified by invasive cancers and DCIS. These components are DNAm-based predictors of circulating plasma protein concentrations, rather than directly measured concentrations. In unadjusted models, five DNAm GrimAge components were associated with breast cancer (Table 2). Specifically, DNAm-predictors of adrenomedullin (per one-SD increase: HR: 1.25, 95% CI: 1.13, 1.39, *P*<0.001); cystatin C (HR: 1.71, 95% CI: 1.50, 1.95, *P*<0.001); growth differentiation factor-1 (HR: 1.48, 95% CI: 1.29, 1.69, *P*<0.001); leptin (HR: 1.13, 95% CI: 1.05, 1.23, *P*=0.002); and tissue inhibitor metalloproteinase 1 (HR: 1.69, 95% CI: 1.45, 1.96, *P*<0.001). After adjustment for breast cancer risk factors, associations remained for the DNAm-predictors of cystatin C (HR: 1.37 95% CI: 1.16, 1.62, *P*<0.001) and leptin (HR: 1.12 95% CI: 1.03, 1.21, *P*=0.01). Similar patterns were observed for invasive breast cancer, although associations with DCIS tended to be weaker.

**Table 2:** DNAm GrimAge components and breast cancer risk overall and stratified by stage at diagnosis.

**GrimAge component**	**All breast cancer (DCIS and invasive combined)**		**Invasive breast cancer**		**Ductal carcinoma *in situ***	
	**HR (95% CI)**	**P-value**	**HR (95% CI)**	**P-value**	**HR (95% CI)**	**P-value**
Adrenomedullin						
Model 1	1.25 (1.13, 1.39)	< 0.001	1.30 (1.16, 1.45)	< 0.001	1.10 (0.93, 1.30)	0.27
Model 2	1.05 (0.93, 1.18)	0.41	1.09 (0.96, 1.23)	0.20	0.93 (0.77, 1.13)	0.49
Beta-2-microglobulin						
Model 1	1.11 (0.99, 1.24)	0.08	1.09 (0.96, 1.23)	0.17	1.19 (0.98, 1.45)	0.08
Model 2	0.91 (0.79, 1.04)	0.15	0.87 (0.76, 1.01)	0.07	1.02 (0.81, 1.29)	0.87
Cystatin C						
Model 1	1.71 (1.50, 1.95)	< 0.001	1.73 (1.51, 1.99)	< 0.001	1.62 (1.32, 1.99)	< 0.001
Model 2	1.37 (1.16, 1.62)	< 0.001	1.38 (1.16, 1.65)	< 0.001	1.32 (1.00, 1.75)	0.05
GDF-15						
Model 1	1.48 (1.29, 1.69)	< 0.001	1.50 (1.30, 1.73)	< 0.001	1.42 (1.12, 1.80)	0.003
Model 2	1.05 (0.87, 1.26)	0.61	1.06 (0.88, 1.28)	0.55	1.01 (0.72, 1.40)	0.97
Leptin						
Model 1	1.13 (1.05, 1.23)	0.002	1.18 (1.08, 1.28)	< 0.001	0.99 (0.87, 1.12)	0.84
Model 2	1.12 (1.03, 1.21)	0.009	1.15 (1.06, 1.26)	0.002	1.00 (0.87, 1.14)	0.97
PAI-1						
Model 1	1.06 (0.98, 1.15)	0.17	1.09 (1.00, 1.19)	0.04	0.94 (0.82, 1.07)	0.36
Model 2	0.97 (0.88, 1.07)	0.57	0.99 (0.90, 1.10)	0.89	0.90 (0.76, 1.06)	0.22
TIMP-1						
Model 1	1.69 (1.45, 1.96)	< 0.001	1.67 (1.42, 1.95)	< 0.001	1.75 (1.36, 2.26)	< 0.001
Model 2	0.95 (0.74, 1.23)	0.72	0.91 (0.70, 1.20)	0.51	1.12 (0.73, 1.72)	0.61
Smoking pack-years						
Model 1	1.06 (0.98, 1.14)	0.17	1.06 (0.98, 1.15)	0.15	1.04 (0.91, 1.18)	0.60
Model 2	1.02 (0.93, 1.11)	0.68	1.02 (0.93, 1.12)	0.64	1.00 (0.87, 1.15)	0.99

Abbreviations: growth differentiation factor, GDF; plasminogen activator inhibitor, PAI; tissue inhibitor metalloproteinase, TIMP.

Model 1: Crude, unadjusted. (Events/at risk: overall, 1,569/2,773; Invasive, 1,231/2,449; DCIS, 338/1,618)

Model 2: Adjusted age at enrollment plus baseline status of body mass index (BMI), menopause, a BMI-menopause interaction term, physical activity, alcohol intake, parity, age at first birth (among parous), age at menarche, breastfeeding duration, and hormone therapy and oral contraception duration (Events/at risk: overall, 1,550/2,727; Invasive, 1,216/2,407; DCIS, 334/1,586)

Abbreviations: hazard ratio, HR; confidence interval, CI; ductal carcinoma in situ, DCIS.

## DISCUSSION

Using a nationwide, prospective cohort designed to identify novel breast cancer risk factors, we found a weak association between GrimAgeAccel and invasive breast cancer. Further stratification of invasive breast cancers by tumor ER status and menopause status at diagnosis revealed somewhat enhanced associations between GrimAgeAccel and incidence of ER-positive and postmenopausal breast cancers. Although our findings suggest that this epigenetic mortality predictor may be a weak marker for these specific breast cancer subtypes, in post-hoc analyses, we found that some of the individual components of DNAm GrimAge appeared to have stronger associations with breast cancer. These components are DNAm-based predictions of circulating plasma proteins and are not based on direct measurements in study subjects. Despite the moderate correlation with GrimAgeAccel, the MS was not related to breast cancer incidence.

Although GrimAgeAccel and the MS were both designed to predict all-cause mortality, we only observed breast cancer associations with GrimAgeAccel. Differences in the design of these two mortality predictors may explain the different associations: unlike the MS, DNAm GrimAge did not directly select mortality-associated CpGs. Instead, DNAm GrimAge is based on CpGs that are predictive of mortality-associated risk factors, specifically, smoking pack-years and various plasma proteins [26]. Interestingly, the DNAm-predictors of leptin and cystatin C that are components of DNAm GrimAge were strongly associated with breast cancer incidence, even after adjustment for breast cancer risk factors. These DNAm predictors were developed in the FHS using elastic net regularization to select sets of CpGs that correlate with the plasma protein level. Although research on cystatin C and breast cancer is limited, prospective studies suggest leptin might be a marker of breast cancer risk [30, 31]. Thus, the association between GrimAgeAccel and breast cancer may be partly due to the inclusion of CpGs that correlate with plasma concentrations of these proteins.

Both epigenetic mortality predictors include CpGs previously reported to be associated with past smoking behaviors [26, 27], a strong risk factor for mortality [32]. GrimAgeAccel includes 1,030 CpGs, of which 172 (17%) are associated with self-reported pack-years [26]. Similarly, of the ten CpGs included in the MS, 40% have associations with smoking history [27, 33]. Smoking history is an important predictor of all-cause mortality but has little influence on breast cancer incidence [34]. Although the inclusion of smoking-related CpGs may enhance associations with smoking- related cancers [35], such CpGs likely offer little benefit in predicting cancer where smoking does not play a role. In this study, we did not observe associations between the DNAm-predictor of smoking pack-years and breast cancer, providing additional support that CpGs related to smoking history may not be informative for predicting breast cancer.

While we focused on unadjusted association estimates for the epigenetic mortality predictors, we found that adjustment for breast cancer risk factors attenuated associations. Although unadjusted analysis is appropriate given that we were primarily interested in assessing predictive utility of the epigenetic mortality predictors, the attenuation of the associations suggests the GrimAgeAccel metric may only be a marker of breast risk factors, rather than having independent associations with disease risk. Conversely, associations for the DNAm-predictors of cystatin C and leptin were robust to adjustment and may be useful DNAm-based markers for predicting breast cancer. Notably, in the FHS where these markers were developed, the DNAm-predictors of these proteins only had moderate correlations with directly measured concentrations (cystatin C: ρ= 0.39; leptin: ρ= 0.35) [26]. Another consideration is that breast cancer is a heterogenous disease with different etiologies [36–38]; our findings suggest GrimAgeAccel may only be associated with postmenopausal and ER-positive invasive tumors. Interestingly, these tumors are the most prevalent and have good survival [28]. It is likely that we were underpowered to test associations with the less prevalent, more lethal subtypes—fewer than 200 women were diagnosed with either premenopausal or ER-negative breast cancers. The epigenetic mortality predictors may also be better markers for the incidence of late stage breast cancer or breast cancer mortality but only 98 (5%) women in our sample were diagnosed with stage III or IV disease and overall survival is quite high [28]. This is, however, the largest available study to examine blood methylation predictors of breast cancer; designing future studies to investigate blood DNAm predictors of breast cancer in more diverse populations that have higher incidences of aggressive phenotypes may be required.

In summary, we find that the GrimAgeAccel mortality predictor was weakly associated with postmenopausal and ER-positive invasive breast cancers. Given the reliance on smoking-associated CpGs, it is perhaps unsurprising that the associations with breast cancer incidence were weak. Other components of DNAm GrimAge, however, appeared to be useful in predicting breast cancer. Blood DNAm may be a useful matrix to derive novel breast cancer predictors. For example, epigenetic clocks were designed to capture age effects and are associated with breast cancer incidence [25]. Single CpGs are also associated with breast cancer incidence [39, 40] and may be sensitive to emerging and established breast cancer risk factors [41–46]. Future efforts to design epigenetic breast cancer predictors may therefore be aided by selecting CpGs associated with both breast cancer risk factors as well as the disease itself.

## MATERIALS AND METHODS

### Sample population

The Sister Study is a prospective cohort of 50,884 cancer-free women, recruited from the United States (including Puerto Rico) and enrolled between 2003–2009 [47]. Eligible women were between the ages of 35 and 75, could not have breast cancer themselves, but had a biological sister previously diagnosed with the disease. Participants are re-contacted annually to update information on breast cancer and response rates are approximately 95%. Among the women who report an incident breast cancer, permission to retrieve medical records is requested six months after diagnosis. The positive predictive value of a self-reported breast cancer in this population is approximately 99.4% [48]. In July 2014, a case-cohort subsample of non-Hispanic white women was selected for blood genome-wide DNA methylation analysis [49]. This subsample included 1,294 women randomly selected from the full cohort, of whom 90 developed incident breast cancer (invasive or ductal carcinoma *in situ*), and 1,479 additional women who developed breast cancer after blood draw (data release 6.0). Whole blood samples and informed consent were obtained at a home visit. The institutional review boards at the National Institute of Environmental Health Sciences and the Copernicus Group approved the study.

### Genomic DNA processing and epigenetic mortality predictor calculation

Processing procedures for the DNA samples have been previously reported [50]. Genomic DNA from whole blood samples was extracted using DNAQuick at BioServe Biotechnologies LTD (Beltsville, MD) or using an automated system (AutoPure LS, Gentra Systems) in the NIEHS Molecular Genetics Core Facility. One microgram of extracted DNA was bisulfite-converted using the EZ DNA Methylation kit (Zymo Research, Orange County, CA). After testing for complete bisulfite conversion, following the manufacturer’s protocol, DNA was analyzed using Illumina’s Infinium HumanMethylation450 BeadChip. To reduce batch effects, arrays were processed using high throughout robotics. Methylation analysis was conducted at the NIH Center for Inherited Disease Research (Baltimore, MD).

The *Enmix* R software package was used for methylation data preprocessing and quality control [51]. This included background noise reduction using the ENmix method; applying the RELIC method to correct for fluorescent dye-bias; quantile normalization to make overall fluorescence intensity distribution comparable between arrays; and reducing probe design bias using the ‘regression on correlated probes’ method [52]. Data from the Sister Study can be requested via https://sisterstudy.niehs.nih.gov/English/coll-data.htm. GrimAgeAccel was calculated using an online calculator (https://dnamage.genetics.ucla.edu/home) and a continuous version of the MS was calculated as described by the developers [27].

### Statistical analysis

Although GrimAgeAccel was designed to be independent of chronological age, the MS was not. We therefore regressed the MS on chronological age and predicted the residuals to create a MS that was independent of chronological age to use in our main analyses. We assessed Pearson correlations between the epigenetic mortality predictors and chronological age. We standardized the epigenetic mortality predictors and the individual DNAm GrimAge components to have means of zero and standard deviations of one. To examine associations with breast cancer risk, we used case-cohort Cox proportional hazard models to calculate hazard ratios, 95% confidence intervals and *P*-values. We treated chronological age as the time-scale in all models. For the primary analysis, we combined invasive and ducal carcinoma *in situ* to represent breast cancer overall. In secondary analyses, we considered those categories separately. We also investigated associations for invasive breast cancer by menopausal status at diagnosis and tumor estrogen receptor status. Because we were interested in assessing predictive utility of these biomarkers, we focused on unadjusted associations. However, we also examined associations accounting for established breast cancer risk factors, including: body mass index (BMI), menopause, a BMI-menopause interaction term, physical activity, alcohol intake, parity, age at first birth (among parous), age at menarche, breastfeeding duration, and hormone therapy and oral contraception duration [37, 38, 53–57]. All analyses were conducted using Stata version 15 (College Station, TX).

## Supplementary Material

Supplementary Figures

Supplementary Tables
