# Supplementary material for: Epigenetic mortality predictors and incidence of breast cancer
Source: Aging (Albany NY). 2019 Dec 17;11(24):11975–87. doi: 10.18632/aging.102523 (PMC6949084; doi:10.18632/aging.102523)
Supplement: Supplementary Figures [file aging-11-102523-s002..pdf]

SUPPLEMENTARY FIGURES

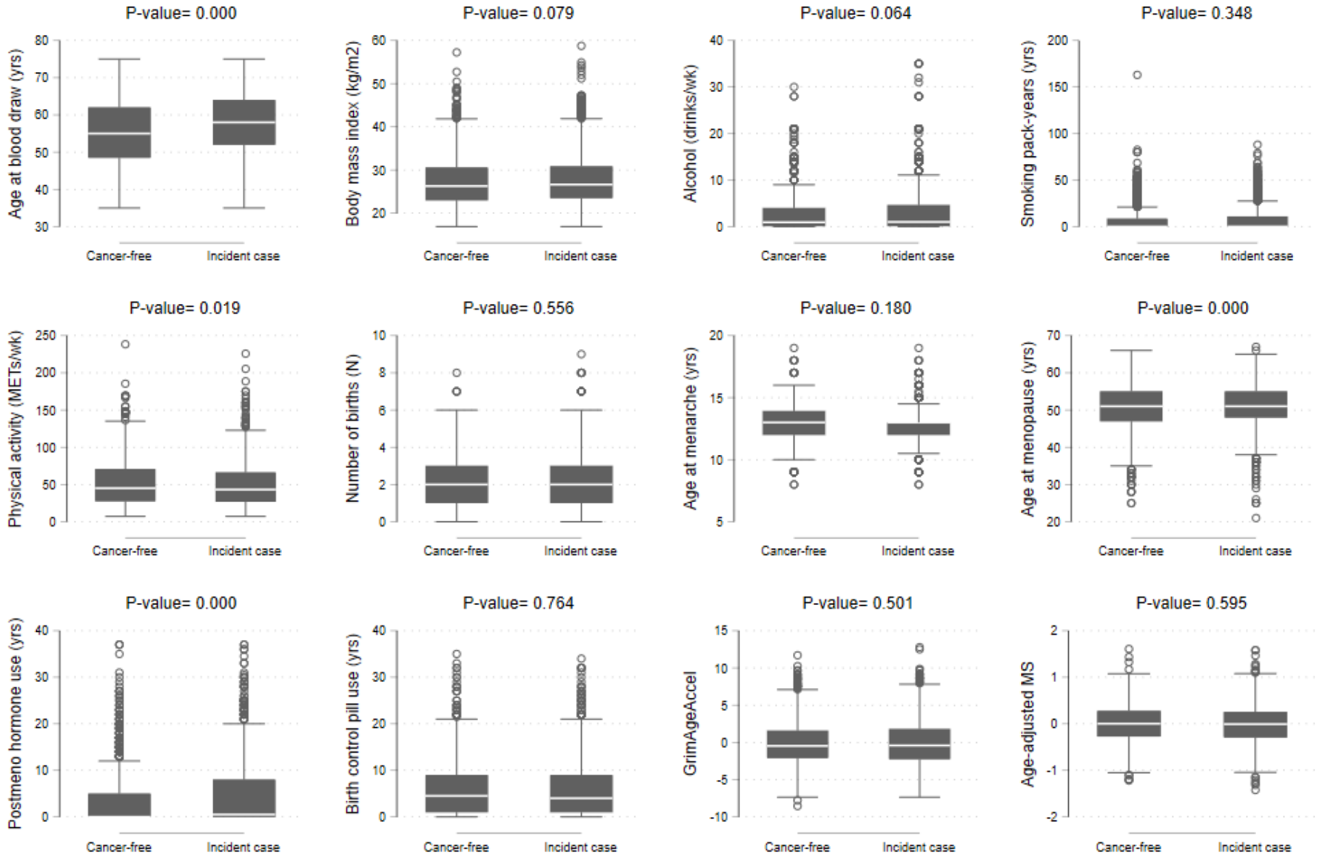

Supplemental Figure 1. Continuous participant characteristics at study enrollment by cancer status at follow-up. Two-sided P-values were calculated using two-sample t-tests.

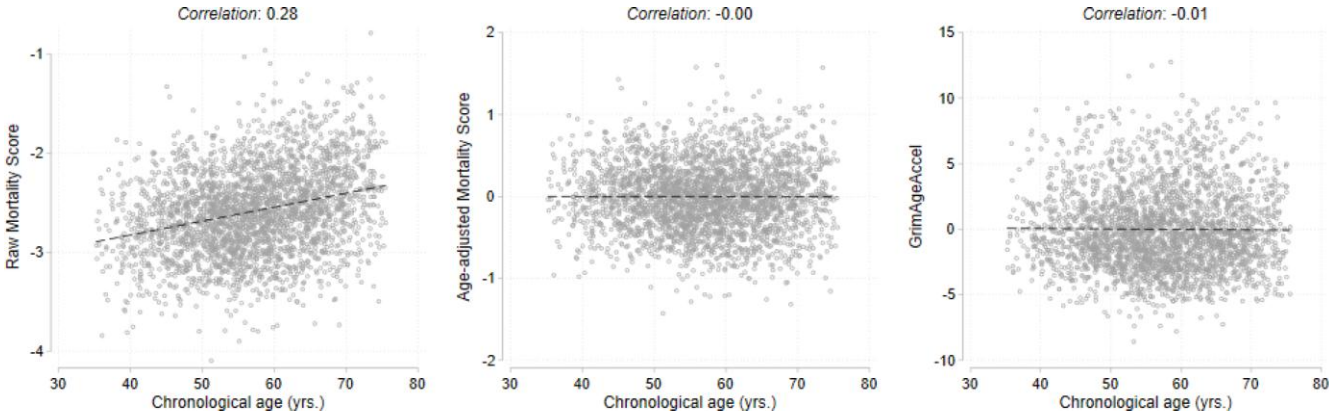

Supplemental Figure 2. Pearson correlations for chronological age and the raw Mortality Score, the age-adjusted Mortality Score and the GrimAgeAccel metric.
