# Supplementary material for: Epigenetic mortality predictors and incidence of breast cancer
Source: Aging (Albany NY). 2019 Dec 17;11(24):11975–87. doi: 10.18632/aging.102523 (PMC6949084; doi:10.18632/aging.102523)
Supplement: Supplementary Tables [file aging-11-102523-s001..pdf]

SUPPLEMENTARY TABLES

Supplementary Table 1. Epigenetic mortality predictors and breast cancer risk overall and stratified by stage at diagnosis.

Mortality predictor	All breast cancer (DCIS & invasive combined)		Invasive breast cancer		Ductal carcinoma *in situ*	
	HR (95% CI)	P-value	HR (95% CI)	P-value	HR (95% CI)	P-value
GrimAgeAccel						
Model 1	1.06 (0.98, 1.14)	0.17	1.08 (0.99, 1.17)	0.08	0.99 (0.86, 1.13)	0.85
Model 2	1.03 (0.95, 1.12)	0.46	1.04 (0.95, 1.14)	0.41	1.01 (0.87, 1.17)	0.95
Mortality Score						
Model 1	0.99 (0.92, 1.07)	0.85	0.98 (0.91, 1.06)	0.65	1.03 (0.91, 1.17)	0.61
Model 2	0.98 (0.90, 1.06)	0.55	0.96 (0.88, 1.04)	0.30	1.05 (0.92, 1.21)	0.45

Model 1: Crude, unadjusted. (Events/at risk: overall, 1,569/2,773; Invasive, 1,231/2,449; DCIS, 338/1,618)
Model 2: Adjusted age at enrollment plus baseline status of body mass index (BMI), menopause, a BMI-menopause interaction term, physical activity, alcohol intake, parity, age at first birth (among parous), age at menarche, breastfeeding duration, and hormone therapy and oral contraception duration (Events/at risk: overall, 1,550/2,727; Invasive, 1,216/2,407; DCIS, 334/1,586)
Abbreviations: hazard ratio, HR; confidence interval, CI; ductal carcinoma *in situ*, DCIS.

Supplementary Table 2. Epigenetic mortality predictors and invasive breast cancer risk and stratified by menopause at diagnosis or tumor estrogen receptor status.

Mortality predictor	Postmenopausal breast cancer		Premenopausal breast cancer	
	HR (95% CI)	P-value	HR (95% CI)	P-value
GrimAgeAccel				
Model 1	1.10 (1.01, 1.20)	0.04	0.95 (0.79, 1.15)	0.62
Model 2	1.05 (0.95, 1.16)	0.30	1.01 (0.82, 1.24)	0.92
Mortality Score				
Model 1	0.99 (0.91, 1.08)	0.82	0.94 (0.77, 1.14)	0.52
Model 2	0.97 (0.88, 1.06)	0.46	0.97 (0.78, 1.20)	0.78
**Mortality predictor**	**Estrogen receptor positive**		**Estrogen receptor negative**	
	**HR (95% CI)**	**P-value**	**HR (95% CI)**	**P-value**
GrimAgeAccel				
Model 1	1.08 (0.99, 1.17)	0.09	1.03 (0.87, 1.23)	0.74
Model 2	1.04 (0.95, 1.14)	0.40	0.99 (0.81, 1.20)	0.88
Mortality Score				
Model 1	0.98 (0.90, 1.07)	0.62	0.98 (0.84, 1.16)	0.84
Model 2	0.96 (0.87, 1.05)	0.32	0.95 (0.80, 1.13)	0.55

Model 1: Crude, unadjusted. (Events/at risk: postmenopausal, 1,020/2,093; premenopausal, 198/590; ER-positive,1,043/2,270; ER-negative, 168/1,446)
Model 2: Adjusted age at enrollment plus baseline status of body mass index (BMI), menopause, a BMI-menopause interaction term, physical activity, alcohol intake, parity, age at first birth (among parous), age at menarche, breastfeeding duration, and hormone therapy and oral contraception duration (Events/at risk: postmenopausal, 1,005/2,056; premenopausal, 198/583; ER-positive, 1,031/2,231; ER-negative, 165/1,415)
